# High-Fat and Fat-Enriched Diets Impair the Benefits of Moderate Physical Training in the Aorta and the Heart in Rats

**DOI:** 10.3389/fnut.2017.00021

**Published:** 2017-05-18

**Authors:** Cleverson Rodrigues Fernandes, Vinicius Kannen, Karina Magalhães Mata, Fernando Tadeu Frajacomo, Alceu Afonso Jordão Junior, Bianca Gasparotto, Juliana Yumi Sakita, Jorge Elias Junior, Daphne Santoro Leonardi, Fernando Marum Mauad, Simone Gusmão Ramos, Sergio Akira Uyemura, Sergio Britto Garcia

**Affiliations:** ^1^Department of Pathology, University of Sao Paulo, Ribeirao Preto, Brazil; ^2^Department of Toxicology, Bromatology, and Clinical Analysis, University of Sao Paulo, Ribeirao Preto, Brazil; ^3^Department of Internal Medicine, University of Sao Paulo, Ribeirao Preto, Brazil; ^4^Division of Radiology, University of Sao Paulo, Ribeirao Preto, Brazil

**Keywords:** heart, blood vessels, biochemistry, swimming, obesity

## Abstract

**Aim:**

Millions of people die each year due to cardiovascular disease (CVD). A Western lifestyle not only fuses a significant intake of fat with physical inactivity and obesity but also promotes CVD. Recent evidence suggests that dietary fat intake impairs the benefits of physical training. We investigated whether aerobic training could reverse the adverse effects of a high-fat diet (HFD) on the aorta. Then, we explored whether this type of exercise could reverse the damage to the heart that is imposed by fat-enriched diet (FED).

**Methods:**

Rats were randomly assigned to two experiments, which lasted 8 weeks each. First, rats swam for 60 min and were fed either a regular diet [standard diet (STD)] or an HFD. After aortic samples had been collected, the rats underwent a histopathological analysis for different biomarkers. Another experiment subjected rats that were fed either an STD or an FED to swimming for 20 or 90 min.

**Results:**

The first experiment revealed that rats that were subjected to an HFD-endured increased oxidative damage in the aorta that exercises could not counteract. Together with increased cyclooxygenase 2 expression, an HFD in combination with physical training increased the number of macrophages. A reduction in collagen fibers with an increased number of positive α-actin cells and expression of matrix metalloproteinase-2 occurred concomitantly. Upon analyzing the second experiment, we found that physically training rats that were given an FED for 90 min/day decreased the cardiac adipose tissue density, although it did not protect the heart from fat-induced oxidative damage. Even though the physical training lowered cholesterol levels that were promoted by the FED, the levels were still higher than those in the animals that were given an STD. Feeding rats an FED impaired the swimming protocol’s effects on lowering triglyceride concentration. Additionally, exercise was unable to reverse the fat-induced deregulation in hepatic antioxidant and lipid peroxidation activities.

**Conclusion:**

Our findings reveal that an increased intake of fat undermines the potential benefits of physical exercise on the heart and the aorta.

## Introduction

Heart and vascular problems have been categorized as types of cardiovascular disease (CVD), which kills one American every 40 s (1). Recent data have shown that the decrease in CVD burden has decreased from 2011 onward (2). The National Center for Health Statistics reported that heart failure-related deaths increased from 2012 through 2014, although heart failure-related deaths had been reduced from 2000 through 2012 (3). Lessening the incidence of CVD may be an unreachable goal, according to projections from American Heart Association and the Million Hearts Initiative (2).

Physical inactivity and Western diets impair the physiological balance between inflammatory and oxidative stress reactions (4). Dietary fat seems to reduce the systemic antioxidant potential, as it blocks the hepatic production of glutathione (GSH) but induces the oxidation of fatty acids (5, 6). This increase in lipid peroxidation products promotes a chain of events that furthers the development of CVD altering the immune system activity (7–11). For instance, the expression of a known biomarker of inflammation, cyclooxygenase-2 (COX-2), is increased by dietary fat (12). Physiologically, COX-2 exhibits enzymatic activity in endothelial and vascular smooth muscle cells that is known to protect these cells from atherogenesis, although its expression by macrophages promotes CVD (13). Moreover, the authors suggest that macrophages or endothelial cells have a fundamental role from early to late stages of CVD (8, 9, 11), because they can break down collagen by expressing inactive matrix metalloproteinases (MMPs), which are then activated by extracellular proteinases (9, 14). Specifically, the protein complex of membrane type 1 MMP and the tissue inhibitor of MMP2 induce the autocatalytic cleavage of pro-MMP2, thus activating it (14). This may provide a brief overview of how dietary fat causes the tissue remodeling that facilitates the development of CVD (8, 9, 11, 14).

While unhealthy diet, sedentary habits, cigarette smoking, and obesity all act to increase the risk of CVD, previous reports predicted that lifestyle changes may remedy damaged structures in ~80% of cases (1, 15–18). Wisløff and colleagues reported that obese adolescents showed a reduced risk of CVD when they were physically trained twice a week using high-intensity exercises over 3-month period (19). After studying sedentary obese men who were trained using either high-intensity interval or continuous-moderate-intensity physical exercise protocols, Fisher et al. reported that both conditions reduced cardiometabolic risk factors (20). In mice that were fed a high-fat diet (HFD) and were subjected to high-intensity interval physical training, it has been shown that these exercises reduced the diet-induced heart ischemia by improving myocardial oxygenation and diastolic function (16). Pimenta et al. studied the effects of high-intensity interval training on obese female mice that were subjected to an HFD and ovariectomy. They found that the chosen physical training protocol reduced cardiovascular and metabolic risks (17). An interesting report revealed that moderate-intensity exercise for 15 min a day over an 8-year period of time diminished the risk of all-cause mortality, including CVD (21). Another study on middle-aged men further revealed that, rather than resistance exercise training, aerobic Nordic walking for 12 weeks was able to control metabolic syndrome and reduce CVD risk (22). In rats that were fed a standard diet (STD), Suzuki and Machida reported that moderate voluntary aerobic exercise over a period of 35 weeks not only inhibited body weight gain but also reduced CVD risk factors, such as high blood pressure (23). In rats and mice, we found that aerobic exercise promotes health and reduces the inflammatory reaction (24–26).

Although there is currently a great need for new strategies to protect humans who are exposed to Western diets, little is known about whether the benefits of such treatments will be able to surpass the damaging effects of unhealthy lifestyles (15). Notably, Palma-Rigo and colleagues observed that physical training was unable to negate some of the harmful effects of an HFD on the cardiovascular system (27). Additionally, Canet-Soulas and colleagues reported that exercise did not protect against the side effects of high cholesterol intake on cardio-metabolic risk (28).

Here, we sought to explore whether swimming for 1 h five times a week over a period of 56 days could recover to physiological standards some early structural changes in the aorta of rats that were fed an HFD. Given that dietary fat acids worsen cardiac function (29), but that physical training has been proposed to improve it (21, 23), we designed a second experiment. This experiment explored whether physical exercise (swimming for 20 min/day or 90 min/day 5 times/week for 8 weeks) could protect the hearts of rats that were fed fat-enriched diet (FED). Our findings reveal that aerobic training was unable to recover to physiological standards the tissue damage that was promoted by HFD and FED in the aorta and the heart, respectively.

## Materials and Methods

### Animal Management

All experiments followed the guidelines of the Committee on Care and Uses of Laboratory Animals of the National Research Council of the NIH (USA) and were approved by the Committee on Animal Research of the University of São Paulo (#163/2008 and #137/2010). Hence, male Wistar rats (*n* = 80; 60 days) were housed in plastic cages in a controlled room for light–dark cycles (12:12 h), temperature (24 ± 1°C), and humidity (60–70%). Rats had free access to water and standard chow and underwent adaptation to the new environment for 1 week before experiments started.

### Physical Training Protocols

Considering that rats naturally swim in their natural habitat (30), we applied the physical training protocol reported previously by Venditti and Di Meo (31). This strategy reduces animal stress, while physically training them promotes health. Only one researcher of our group was responsible for the animal management and experiments to avoid animals to stress. Because rats were neither subjected to exhaustion nor had any load bound to them while training, the current protocol was considered of moderate intensity (25, 31). According to our previous description (24), rats underwent physical training within a tepid water (32 ± 2°C) in experimental pools (100 cm × 60 cm × 65 cm). Only four rats swam in each pool *per* training period to avoid them to either stay floating or anchor to each other. The swimming pool depths also inhibited rats to rest reaching the bottom with their tail.

### Understanding the Impact of HFD and Exercises on the Aorta—Study I

Here, we randomly divided rats into four groups. Rats given a regular chow (STD; commercial AIN-93 diet, ~4.17 kcal/g) remained sedentary (STD-SED; *n* = 8) or underwent physical training (STD-E60; *n* = 8). Other rats were fed an HFD and remained sedentary (HFD-SED; *n* = 8) or underwent physical training (HFD-E60; *n* = 8). Our research group manufactured that HFD (~6.57 kcal/g) adding 350 g of lard and 200 g of soy oil *per* kg of AIN-93.

Before the experiment started, rats had 7 days for adaptation to the physical training conditions, in which they swam for 15 min five times a week. In the first experimental week, rats swam for 50 min a day. We then added 10 min of exercises to that training period of 50 min in the second experimental week. This training period of 60 min was kept steady for the following 6 weeks.

Rats were euthanized in a CO_2_ chamber at the end of the eighth experimental week. After individual necropsies had been performed, aorta samples were fixed in formalin buffer for 24 h. Formalin-fixed samples were embedded in paraffin before their serial sectioning for histopathological analysis.

### Investigating the Effects of FED and Exercises on the Heart—Study II

This experiment required six groups. Hence, rats given a STD remained sedentary (STD-SED; *n* = 8) or underwent physical training for either 20 min (STD-E20; *n* = 8) or 90 min (STD-E90; *n* = 8). Rats fed a FED remained sedentary (FED-SED; *n* = 8) or underwent physical training for either 20 min (FED-E20; *n* = 8) or 90 min (FED-E90; *n* = 8). Our laboratory synthesized a FED adding ~150g of lard/kg to that commercial AIN-93 chow.

Adaptation to the physical training protocol also lasted 7 days (15-min exercises a day). From the first until the last experimental week, two groups swam for 20 min only (STD-E20 and FED-E20). Other two groups (STD-E20 and FED-E20) exercised for 50 min a day in the 1st experimental week and had this period increased 10 min *per* week until rats swam for 90 min at the fifth experimental week. This 90-min training continued for other 3 weeks. All rats underwent euthanasia at the end of the eighth week. After necropsy, serum and liver samples were frozen liquid nitrogen. The heart was fixed in formalin buffer for 24 h.

### Histopathological Analysis

Paraffin-embedded sections were stained with hematoxylin and eosin (H&E; 5 µm), resorcine (5 µm; it stains elastic fibers), and picrosirius red (7 µm; it stains collagen fibers). After pictures had been taken in a Leica microscopic system (Leica Microsystems GmbH, Germany), we determined in 10 different H&E-stained sections per sample the cardiac [left ventricle (LVT) and right ventricle (RVT), and septum] and the aortic thickness. Elastic and collagen fibers were determined by optical density in eight randomly chosen non-coincident fields at ×40 magnification (values are expressed as %).

Immunohistochemistry was carried out according to our previous description (24). Briefly, tissue sections were exposed to primary antibodies overnight: nitrotyrosine (1/100; ABCAM, USA; AB42789), α-actin (1/100; ABCAM, USA; AB7817), MMP2 (1/100; Santa Cruz Biotechnology, USA; SC-8835), CD68 (1/100; ABCAM, USA; AB31630), and COX-2 [clone 4H12 (1:200); Novocastra, USA]. Nitrotyrosine values are expressed as %, because they were determined by optical density in eight randomly chosen non-coincident fields at ×40 magnification. Then, α-actin, MMP2, CD68, and COX-2 analyses were performed in eight randomly chosen non-coincident microscopic at ×40 magnification. The index was determined to be the number of positive cells per area (square millimeter).

### Adipose Tissue Analysis by Computerized Tomography (CT)

As we previously reported (32), rats from experiment 1 were anesthetized [ketamine (100 mg/kg) and xylazine (10 mg/kg)] and scanned [field (512 mm), thickness (10 mm), intervals (10 mm), 130 kV, 250 mA, 6 s] by the CT [Helical CT Emotion (Siemens, Germany)] in a prone position within a Plexiglass cradle. We applied the MATLAB version 7.0 software (Graphics Software, Natick, MA, USA) for data analysis. It determined the fat density (from −50 to −250 HU; square centimeter) in the liver, heart, and visceral areas.

### Biochemical Analyses

We analyzed total cholesterol (milligrams per deciliter), triglyceride (TG; milligrams per deciliter), aspartate aminotransferase (AST; units per liter), and alanine transaminase (ALT; units per liter) levels in serum samples (33). Hepatic malondialdehyde (MDA; nanomolar per gram) and reduced glutathione (GSH; nanomolar per gram) levels were also determined according to our previous description (32).

### Statistical Analysis

We applied the two-way ANOVA test to analyze all data [Bonferroni’s *post hoc* test; GraphPad Prism 5 (GraphPad Software, USA)]. It enables different categorical independent endpoints to be analyzed on one dependent variable. Because the Bonferroni’s *post hoc* test requires no assumption, we chose it to reduce the chances of differences in current data to be determined significant statistically. A first analysis compared the effects of a physical training within each experimental feeding condition, independently (Tables S1 and S2 in Supplementary Material). The second analysis was performed to clarify how dietary factors might impair the benefits of physical exercises (Tables S3 and S4 in Supplementary Material). Such analysis covered all potential interactions between groups. Statistical significance was set at *p* < 0.05. Shown values are the mean ± SD.

## Results

### A HFD Damages the Aorta despite Benefits from Physical Training

**Figure 1:**
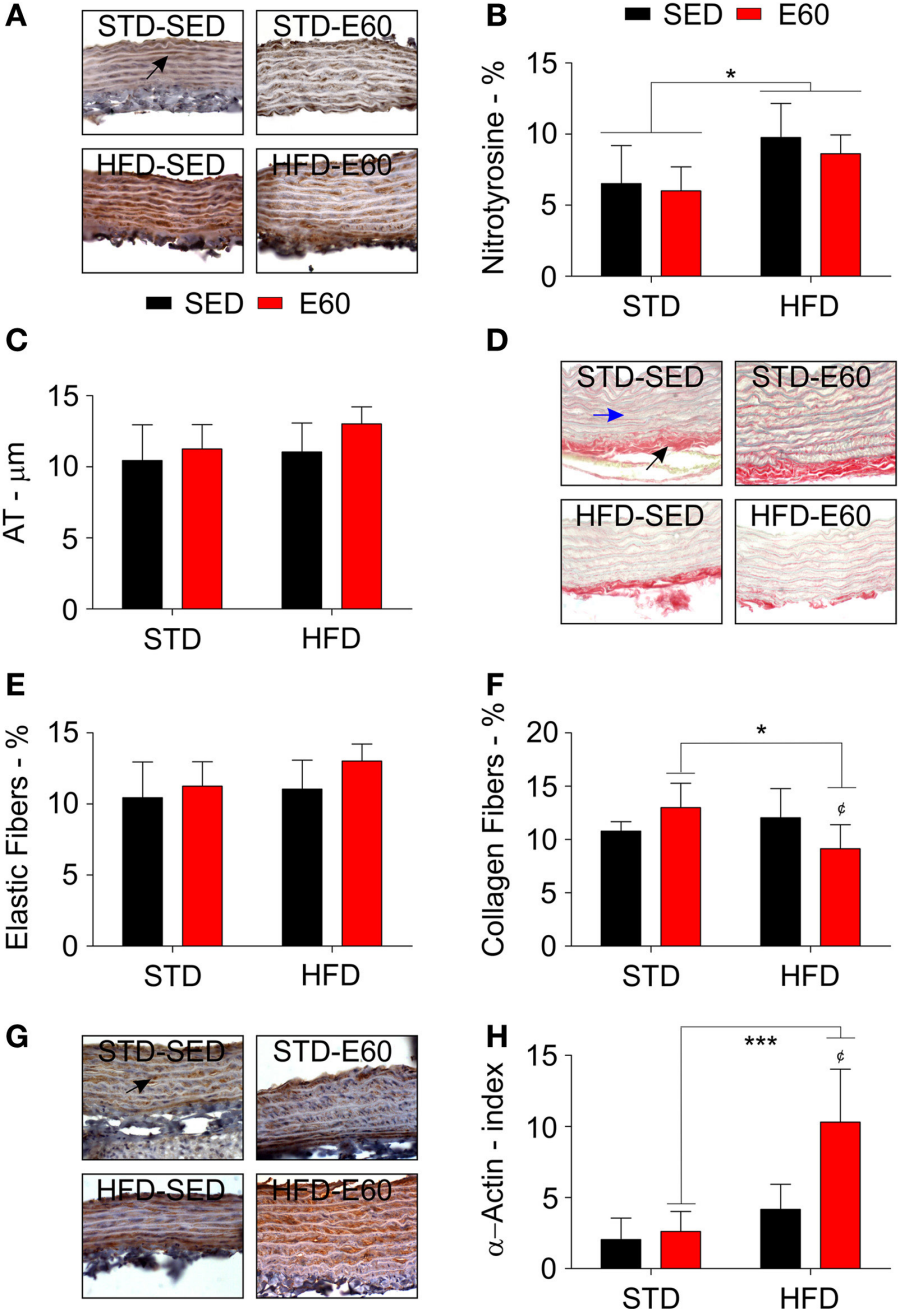
**A high-fat diet (HFD) damages the aorta aside from protective effects of physical training**. **(A)** Representative pictures illustrate reactions with an anti-nitrotyrosine antibody for each experimental group in aorta samples. A black arrow shows a positively stained cell with that anti-nitrotyrosine antibody. Sedentary rats were given a regular chow [standard diet (STD)-SED]; exercised rats were fed a regular chow (STD-E60); sedentary rats were given an HFD (HFD-SED); and, exercised were rats fed an HFD (HFD-E60). **(B)** A histopathological analysis determined nitrotyrosine values (%) by optical density. **(C)** A microscopic analysis determined atrial thickness (micrometers) in aortic slices stained with hematoxylin and eosin. **(D)** Representative pictures illustrate picrosirius red staining for each experimental group in aorta samples. A black arrow shows red-stained collagen fibers. A blue arrow shows yellow stained elastic fibers. **(D,E)** A histopathological analysis determined the density of elastic **(E)** and collagen fibers **(F)**. **(G)** Representative pictures illustrate reactions with an anti-α-actin antibody for each experimental group in aorta samples. A black arrow shows a positively stained cell with that anti-α-actin antibody. **(H)** A microscopic analysis determined the number of positive α-actin cells *per* area (millimeter; index). Statistical Analysis I: ^¢^*p* < 0.05 vs. HFD-SED. Statistical Analysis II: *p < 0.05 and ***p < 0.001. Data analysis: two-way ANOVA test (Bonferroni’s *post hoc* test). Reported values are the mean ± SD.

**Figure 2:**
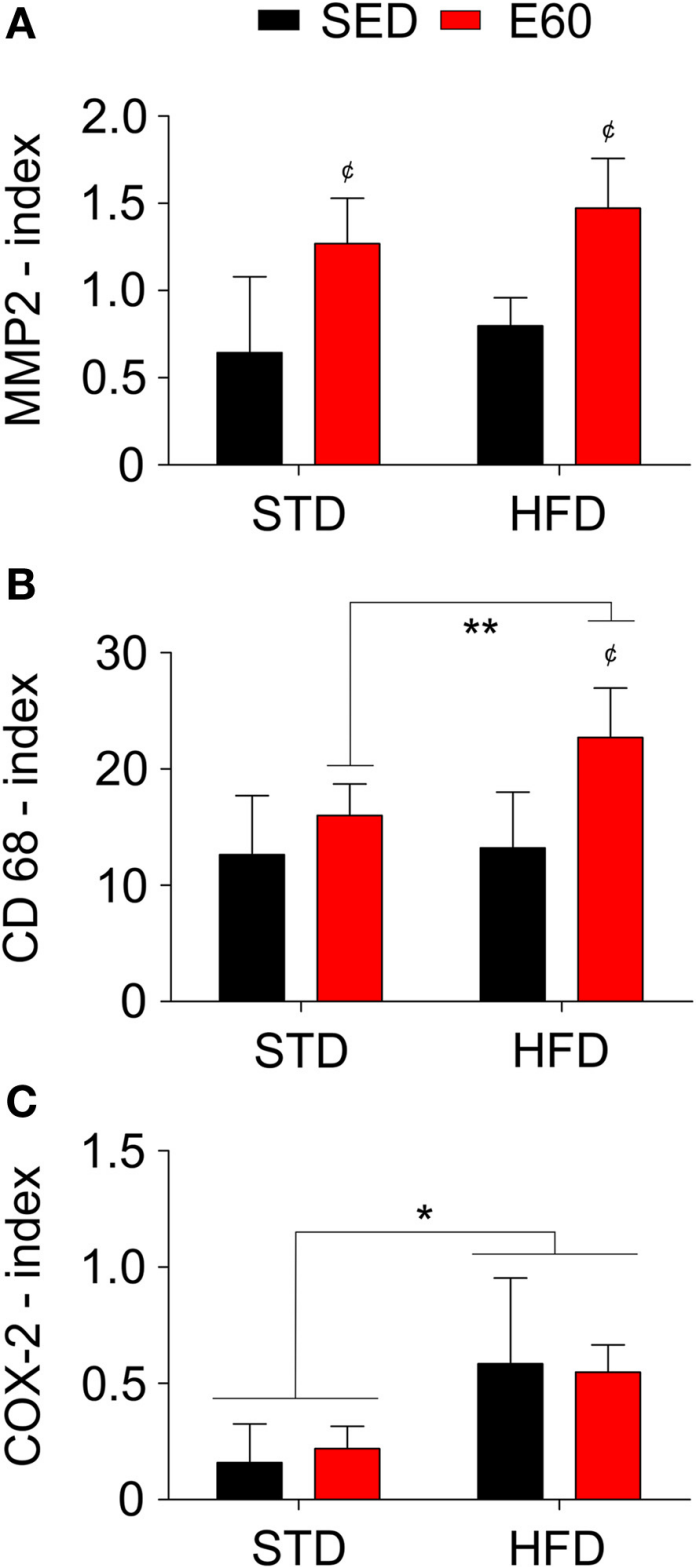
**A high-fat diet (HFD) promotes inflammation in the aortic tissue**. Antibodies against matrix metalloproteinase 2 [MMP2; **(A)**], the cluster of differentiation 68 [CD68; **(B)**], and cyclooxygenase 2 [COX-2; **(C)**] stained aortic samples for histopathological analysis. The index was determined to be the number of positive cells *per* area (square millimeter). Statistical Analysis I: ^¢^*p* < 0.05 vs. STD-SED or HFD-SED. Statistical Analysis II: *p < 0.05 and **p < 0.01. Data analysis: two-way ANOVA test (Bonferroni’s *post hoc* test). Reported values are the mean ± SD.

Considering that dietary fat damages the circulatory system (34), we histopathologically analyzed aorta samples from rats that were fed an HFD but physically exercised for 60 min. The analysis revealed that exercises effectively reduced body weight that had been increased by an HFD (Table 1; *p* < 0.05). By histopathologically analyzing the aorta with a damage biomarker (Figure 1A), we found that physical training was unable to reduce the nitrotyrosine levels that had been increased by an HFD [Figure 1B; *p* < 0.05 (second analysis)]. Other histopathological reactions stained potential tissue structures that dietary fat might damage in the aorta. Neither aorta thickness nor its elastic fiber content was changed in the current experimental conditions (Figures 1C–E; *p* > 0.05). Whereas collagen provides the aorta with mechanical strength (35), we found here that combining physical training with an HFD reduced the aortic content of these fibers [Figure 1F; *p* < 0.05 (first and second analyses)]. Since this fact could be related to tissue remodeling, myofibroblasts were enumerated (36). Thus, we found that physical training increased the number of positive α-actin cells among rats that were fed an HFD [Figures 1G,H; *p* < 0.05 (first analysis); *p* < 0.001 (second analysis)]. We then considered that mesenchymal cells promote MMP2 expression reducing the number of collagen fibers during tissue remodeling (37). Analyzing this metalloproteinase expression revealed that physical exercises augmented its expression in both dietary conditions [Figure 2A; *p* < 0.05 (first analysis)]. Because MMP was shown to be related to aortic remodeling and is expressed by macrophages (37), we enumerated these cells herein. Feeding rats an HFD and physically training them increased the number of positive aortic CD68 cells (Figure 2B; *p* < 0.05). This finding led us to investigate whether inflammation was an event that dietary fat promoted in aortic samples. COX-2 is one of the best biomarkers for determining inflammatory intensity (11, 26). Our findings show that an HFD increased COX-2 expression in the aorta, aside from any positive effects of physical training in rats [Figure 2C; *p* < 0.05 (second analysis)]. Collectively, our data indicate that dietary lipids limit the protective effects of physical training in the aorta.

**Table 1:** **Body weight for each experimental group**.

		Weight (g)				

Groups		Diets		Comparisons		
		STD	HFD			
		Values				
	SED	210.7 ± 15.8	522.8 ± 25.5^#^	^#^*p* < 0.05	*p < 0.05	&*p* < 0.05
Training	E60	190.5 ± 15.5	405.9 ± 18.6^*,#,&^	vs	vs	vs
				STD/SED	HFD/SED	STD/E60

*STD, standard diet; HFD, high-fat diet; SED, sedentary; E60, physical exercises for 60 min*.

*Values are shown as mean ± SD. Two-way ANOVA test (Bonferroni *post hoc* test) was used for all comparisons*.

### Damaging Effects of Enriched-Fat Diet Surpass Benefits of Physical Training on the Heart

**Figure 3:**
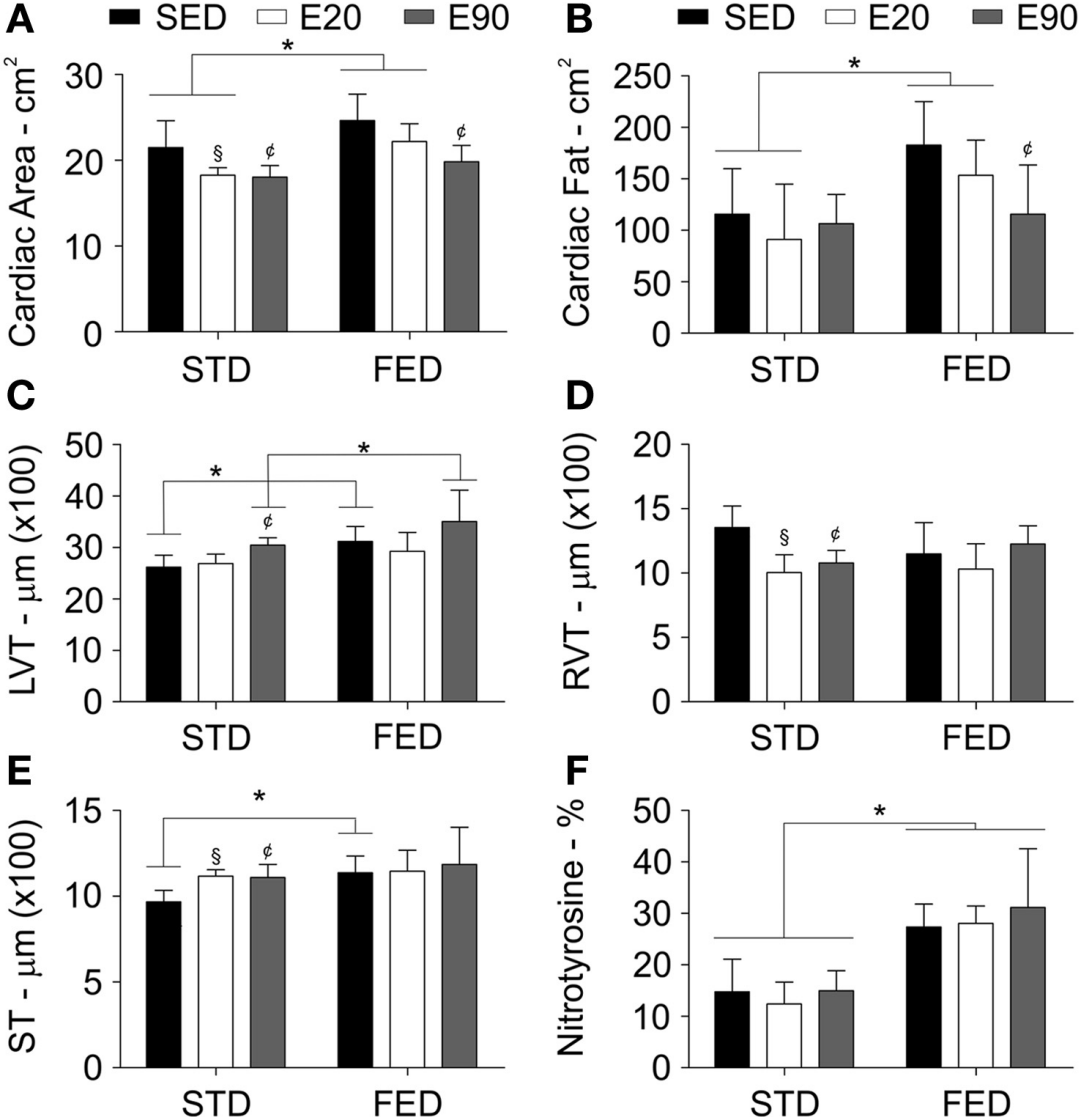
**Physical exercises do not protect the heart from side effects of a fat-enriched diet (FED)**. Computerized tomography analyzed the heart area **(A)**, and the cardiac fat tissue density **(B)**. A histopathological analysis determined the thickness (micrometer) of the left ventricle [LVT; **(C)**], the right ventricle [RVT; **(D)**], and the septum [ST; **(E)**]. Staining heart samples with anti-nitrotyrosine antibody determined damage in this tissue **(F)**. Statistical Analysis I: ^¢^*p* < 0.05 vs STD-SED of FED-SED; ^§^*p* < 0.05 vs STD-SED. Statistical Analysis II: *p < 0.05. Data analysis: two-way ANOVA test (Bonferroni’s *post hoc* test). Reported values are the mean ± SD.

Rather than being exposed to large amounts of dietary fat daily, most modern humans may have a diet best known as FED (38). Despite the aorta being one of the structures that is most damaged by dietary fat, changes in the heart structure should also be carefully considered. Thus, we examined the cardiac area of rats using a CT scan. The whole cardiac area showed a decrease in rats that were physically trained for 20 and 90 min and fed a regular diet [Figure 3A; *p* < 0.05 (first analysis)]. Giving rats an FED increased the cardiac area and adipose tissue density in both the SED and E20 groups [Figures 3A,B; *p* < 0.05 (second analysis)], although regular exercise for 90 min reduced both parameters [Figures 3A,B; *p* < 0.05 (first analysis)].

**Figure 4:**
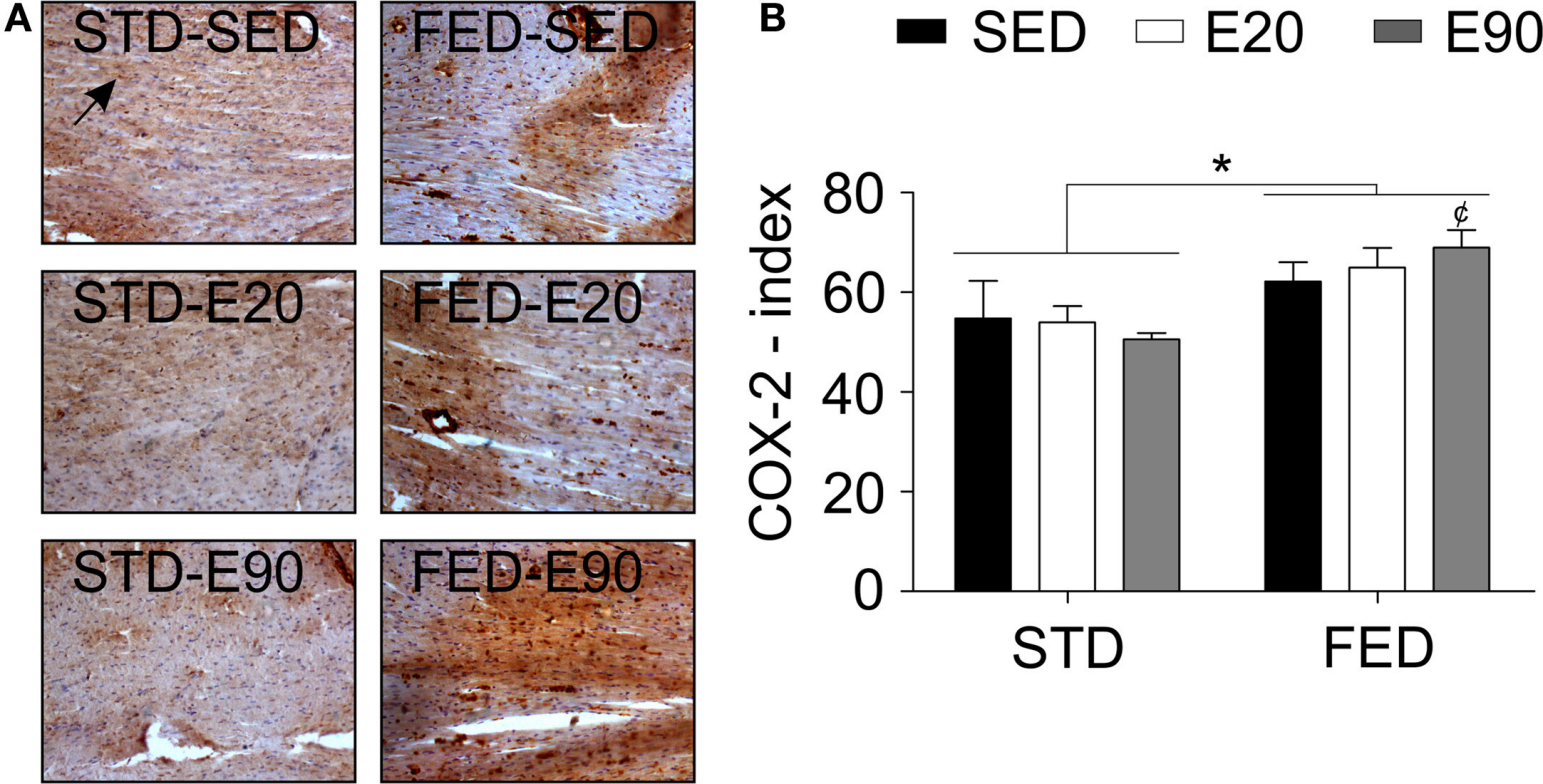
**A fat-enriched diet promotes inflammation in the heart**. **(A)** Representative pictures illustrate reactions with an anti-cyclooxygenase-2 (COX-2) antibody for each experimental group in heart samples. A black arrow shows a positively stained cell with that anti-COX-2 antibody. Sedentary rats were given a regular chow [standard diet (STD)-SED]; exercised rats were fed a regular chow (STD-E60); sedentary rats were given an HFD (HFD-SED); and exercised rats were fed an HFD (HFD-E60). **(B)** The COX-2 index was determined to be the number of positive cells *per* area (square millimeter). Statistical Analysis I: ^¢^*p* < 0.05 *vs*. FED-SED. Statistical Analysis II: *p < 0.05. Data analysis: two-way ANOVA test (Bonferroni’s *post hoc* test). Reported values are the mean ± SD.

A histopathological analysis was then performed to clarify these findings. We found that rats that were given either FED or were physically trained had a thicker LVT [Figure 3C; *p* < 0.05 (first and second analyses)]. Combining exercise (E90) with an FED intake thickened this ventricle even more [Figure 3C; *p* < 0.05 (second analysis)]. Both physical training protocols reduced the RVT thickness, but increased the cardiac septal thickness in rats that were given an STD [Figures 3D,E; *p* < 0.05 (first analysis)]. Feeding rats an FED enlarged the cardiac septum [Figure 3E; *p* < 0.05 (second analysis)]. To determine whether these findings were related to damage of the cardiac tissue, samples were stained with an anti-nitrotyrosine antibody. This analysis showed that an augmented dietary intake of fat damaged cardiomyocytes [Figure 3F; *p* < 0.05 (second analysis)]. In the cardiovascular system, nitrotyrosine and COX-2 cellular expressions have been closely related to inflammation (39). Here, we found that increasing the fatty acid intake promoted COX-2 positive cell numbers in the heart [Figures 4A,B; *p* < 0.05 (second analysis)], a fact that was worsened by exercising rats for 90 min [Figure 4B; *p* < 0.05 (first analysis)]. Careful consideration should be given to the fact that the current physical training protocol was unable to properly protect the heart from the damaging effects of dietary fat.

### FED Worsens the Oxidant/Antioxidant Balance in the Liver

**Figure 5:**
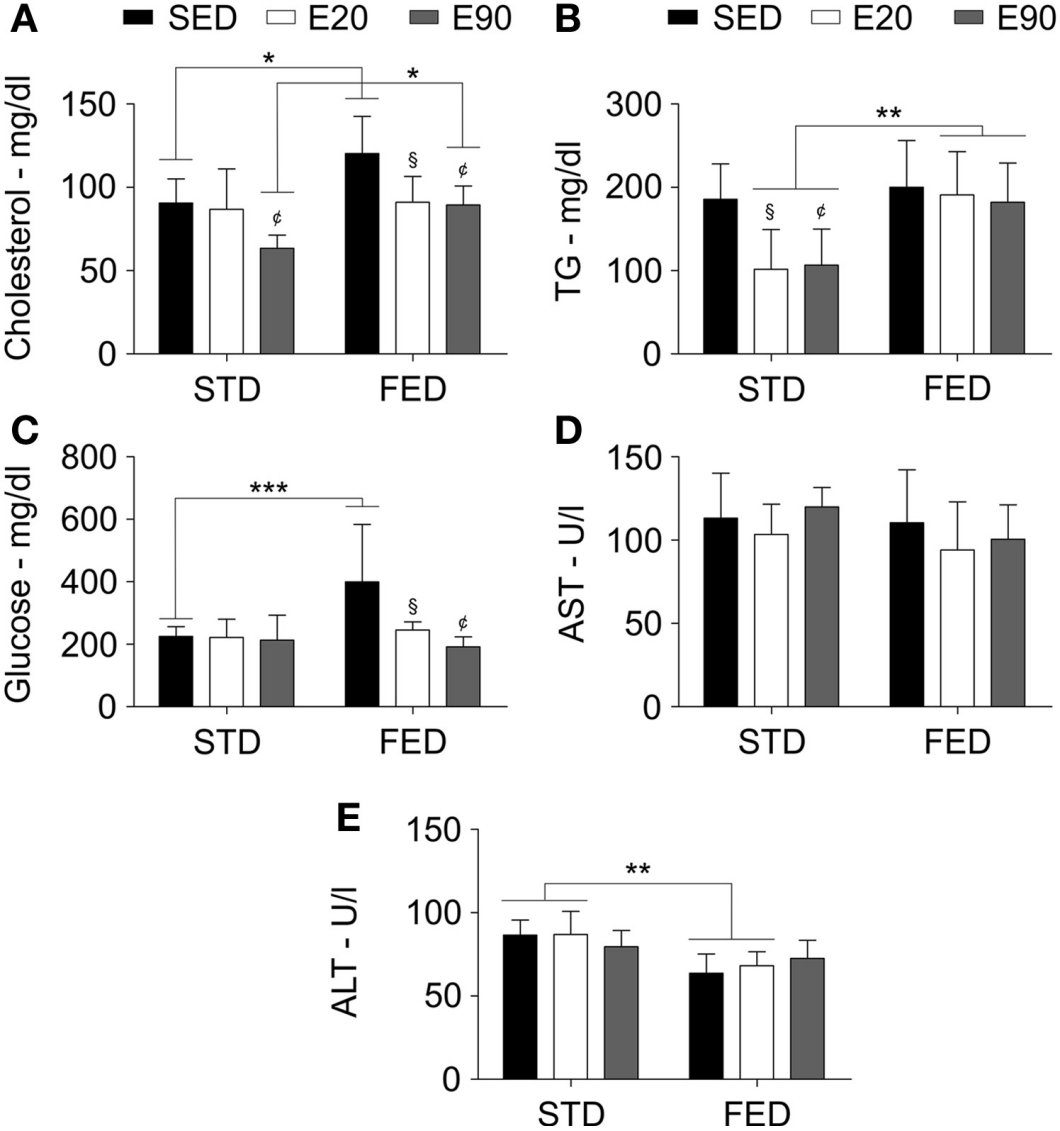
**A fat-enriched diet and physical training alter serum biochemical markers**. Standard biochemical analyses determined serum levels of cholesterol **(A)**, triglycerides [TG; **(B)**], glucose **(C)**, aspartate aminotransferase [AST; **(D)**], and alanine aminotransferase [ALT; **(E)**]. Statistical Analysis I: ^¢^*p* < 0.05 vs standard diet (STD)-SED or FED-SED; ^§^*p* < 0.05 vs STD-SED or FED-SED. Statistical Analysis II: *p < 0.05, **p < 0.01, ***p < 0.001. Data analysis: two-way ANOVA test (Bonferroni’s *post hoc* test). Reported values are the mean ± SD.

The systemic balance between oxidant and antioxidant levels is primarily regulated by the liver and might alter cardiac functions (40). Thus, we analyzed some fat-related serum biomarkers that are classically worsened by increasing lipid intake. Although 90 min of physical training decreased cholesterol serum concentration [Figure 5A; *p* < 0.05 (first analysis)], an FED kept such levels higher than in the SED and E90 groups in comparison with rats that were given regular diet [Figure 5A; *p* < 0.05 (second analysis)]. Physically trained rats that were given an STD had decreased TG levels [Figure 5B; *p* < 0.05 (first analysis)], a benefit that an FED intake impaired [Figure 5B; *p* < 0.01 (second analysis)]. Considering that triglycerides have the bidirectional potential of systemic exchange between fatty acids and glucose from the liver, we then analyzed the serum glucose concentration. Whereas increasing the dietary fat content augmented glucose levels [*p* < 0.001 (second analysis)], both physical training protocols reduced it [Figure 5C; *p* < 0.05 (first analysis)]. None of the current experimental conditions altered the serum levels of the hepatic damaging biomarker, AST (Figure 5D; *p* > 0.05). Interestingly, feeding rats with an FED decreased ALT levels [Figure 5E; *p* < 0.01 (second analysis)].

**Figure 6:**
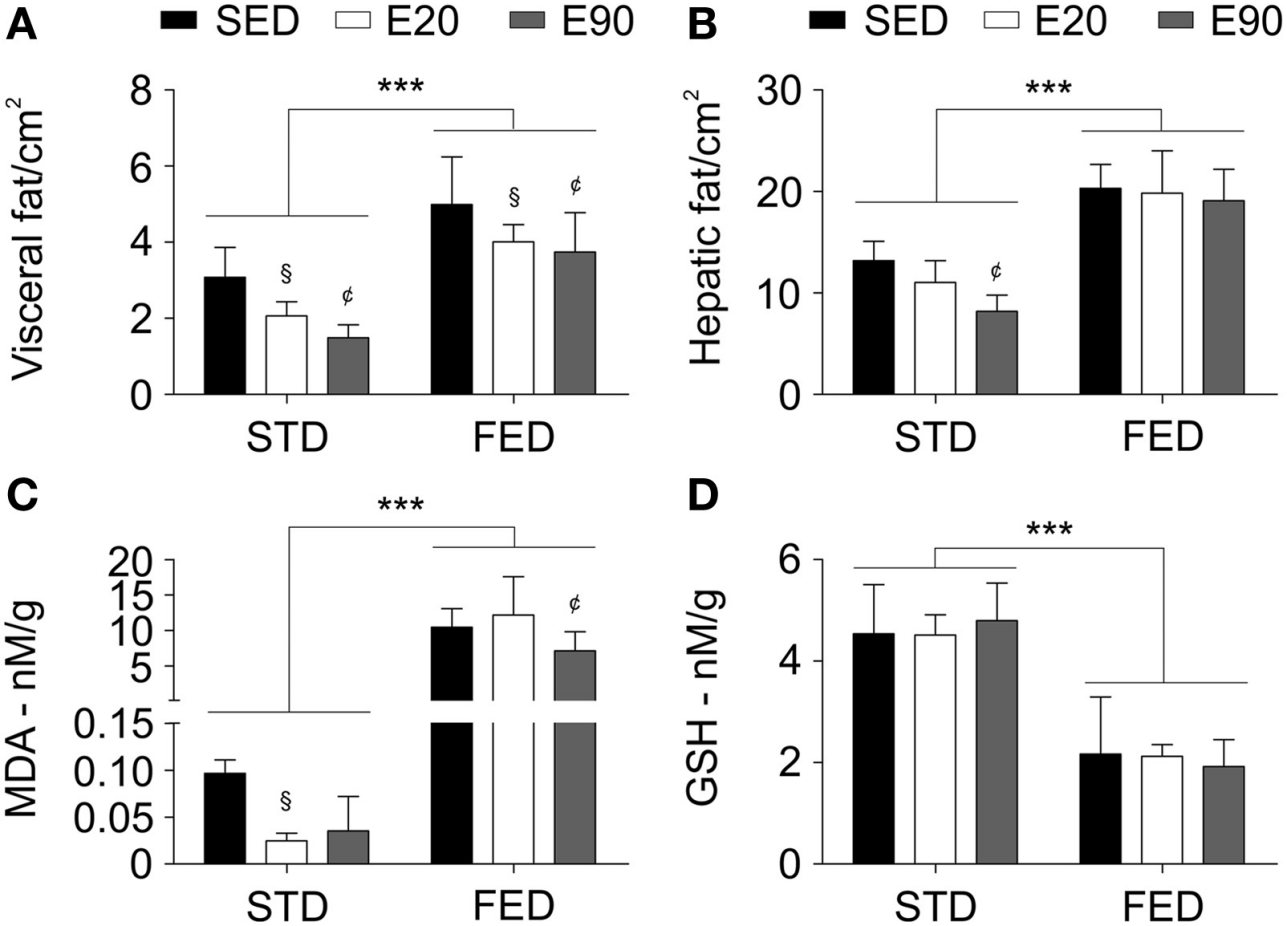
**A physical training does not recover the hepatic tissue damage induced by a fat-enriched diet**. Computerized tomography analyzed the fat tissue density in visceral **(A)** and liver areas **(B)**. Standard biochemical analyses determined malondialdehyde [MDA; **(C)**] and reduced glutathione [GSH; **(D)**] levels in liver samples. Statistical Analysis I: ^¢^*p* < 0.05 vs standard diet (STD)-SED or FED-SED; ^§^*p* < 0.05 vs STD-SED or FED-SED. Statistical Analysis II: ***p < 0.001. Data analysis: two-way ANOVA test (Bonferroni’s *post hoc* test). Reported values are the mean ± SD.

To dissect the fact that increasing the content of dietary fat did not promote changes in a hepatic biomarker of damage, we examined rats using a CT scan to determine the fat density in visceral and liver areas. Increasing the dietary intake of fat augmented the adipose tissue density in visceral organs and the liver [Figures 6A,B; *p* < 0.001 (second analysis)]. Notably, the two physical training protocols reduced fat density in visceral areas in both dietary conditions [Figure 6A; *p* < 0.05 (first analysis)]. Only rats that were fed a regular diet and exercised for 90 min showed reduced fat mass in the liver [Figure 6B; *p* < 0.05 (first analysis)]. Dietary fat seemed to promote lipid peroxidation but inhibit an antioxidant enzyme [Figures 6C,D; *p* < 0.001 (second analysis)]. Although rats that were given STD and were physically trained for 20 min showed reduced lipid peroxidation [*p* < 0.05 (first analysis)], only 90 min of exercises decreased this process in the liver in animals that were subjected to an FED [Figure 6C; *p* < 0.05 (first analysis)]. Interestingly, physical training did not improve the antioxidant hepatic potential (Figure 6D; *p* > 0.05). Our findings reveal that slight changes in dietary fat intake impair the balance between lipid peroxidation and antioxidant capacity in the liver.

## Discussion

We initially planned to reveal potential benefits to the aorta and the heart from aerobic physical exercise that previous reports have shown to be protective against a life-threatening condition (24–26, 41). The following discussion will dissect the matter of increasing the dietary fatty intake, which may impair the benefits of a physical training protocol on the aorta and the heart.

### Understanding the Impact of HFD and Exercises on the Aorta

First, we highlight that physical training reduced the HFD-induced increase in body weight gain ~1.3-fold, although this particular diet more than doubled the weight of the animals. Basic calculations of weight gain reveal that these increments of dietary fat increased weight by approximately 2.3-fold in both sedentary and exercised rats. These facts could be likened to the null-protective effect of physical activity on aortic damage. In mice that were fed an HFD for up to 6 months, Tan and colleagues showed that fat elicits aortic remodeling through the inflammatory response in a time-dependent manner (34). Indeed, our investigation adds strength to the idea that fat oxidatively damages the aorta, since current findings were quite similar to this previous report, but occurred after a much shorter period of dietary exposure. It seems that blocking the protective nitric oxide reaction in the aorta promotes peroxynitrite formation, which has nitrotyrosine as its footprint (42). Stephen et al. reported that oxidative events also alter the behavior of collagen fibers, increasing their stiffness (43). Dietary fat that increases lipid peroxidation might also nurture the aortic remodeling through enhanced inflammatory reaction (44).

Further, in the physically trained rats that were fed an HFD, we found that a 1.2-fold increase in aortic thickness occurred along with signs of matrix remodeling. This may be better understood considering that expression of MMP2, α-actin, COX-2, and CD68 increased together, with a reduced number of collagen fibers. Thus, dietary fat seems to alter the aortic structure in a chain of events that is related to its deregulatory effects on the immune system. Recently, another research group found that lipid oxidation might be linked to an aortic aneurysm since it could promote elastic fibers degradation and high-MMP2 expression (45). Experiments using double-knockout mice (ApoE/iNOS) that were fed an HFD showed that cholesterol induces atheromas, not only reducing collagen content but also increasing the expression of MMP in an inducible nitric oxide synthase-dependent manner (7). Furthermore, lipid peroxidation largely altered macrophage activity, as it reduced their expression of tissue inhibitors of MMP3 and promoted inflammation and the formation of atherosclerotic plaques (46). Schiffrin and colleagues then showed that an HFD induces endothelial cells to express endothelin-1 leading to proinflammatory signaling, which recruits monocytes to the atherosclerotic sites and promotes their differentiation into macrophages (9). During initial atherosclerotic steps, blood leukocytes bind to activated endothelial cells and migrate toward the intima cellular layer, a step that enables their maturation into macrophages and later, their transformation into foam cells through high-lipid uptake (11). From our dataset, we noticed that combining HFD with exercise enlarged the colonic macrophage population, while inflammation (COX-2 index) was increased solely by an HFD. We must observe that Kishimoto and colleagues found that swimming did not reduce the severity of experimental atherosclerosis promoted by an HFD unless it was associated with an inhibitor of nitric oxide synthase, named NG-nitro-l-arginine methylester (47). Interestingly, aspirin reduced COX-2 expression and macrophage numbers in atheromas from rabbits that were given cholesterol (8). Treating mice with selective COX-2 inhibitors revealed that AMPKα1 activity promoted this inflammatory signaling in endothelial cells (10).

### Investigating the Effects of FED and Exercises on the Heart

Given that the total dietary fat content was ~3.6-fold less in an FED than in an HFD and that 60 min of exercise did not reduce damage to the aorta, we sought to adjust the daily time of physical training to be either one-third less than 60 min (E20) or one-third more (E90) in rats fed with an FED. Daily 90-min exercise recovered to standard levels the cardiac area and adipose tissue density that was enlarged by an FED, despite the fact that dietary changes thickened the LVT. In 1977, Valli and colleagues reported that rats given lard endured increased heart weight along with high-lipid density in the heart (48). Approximately 35 years later, mice that were exposed to increased dietary fat content showed left ventricular hypertrophy (49). Moreover, such diets promoted shrinkage in systolic diameter (50). Although we observed that exercised rats that were fed a regular diet had a slight trend of decreased inflammation in the cardiac tissue, fatty acids seemed to promote damage-related inflammatory reactions in both the aorta and the heart. A study on heart transplantation in rats helps to clarify that inflammation damages cardiomyocytes (51). Thus, the authors reported that rats that were treated with a selective COX-2 inhibitor showed reduced cardiac allograft rejection and myocardial damage (51).

Despite the fact that FED-induced cardiac changes were not counteracted by exercises, we observed that physical training reduced serum cholesterol and glucose levels but did not alter TG concentration. Thus, we should remember that an increased fat intake alters the mitochondrial bioenergetics, blocking the hepatic and skeletal muscle uptake of glucose, which then augments its serum levels (49, 52). Interestingly, a report revealed that short-term voluntary running for 2 weeks promoted the insulin activity, improving the muscle-related glucose transport in mice (53). These data drove our attention toward hepatic metabolism. We found that exercise reduced the fat-induced hepatic lipid peroxidation by 1.5-fold, while an FED still promoted oxidation of fatty acids 270-fold more than was found in rats that were subjected to an STD. Since hepatic GSH concentration was lowered 2.2-fold, we should not forget that the hepatic fat density doubled following the exposure to an FED. Non-alcoholic fatty liver disease decreases hepatic TG clearance and antioxidant capacity, whereas fat deposition increases (6). However, we clarify that our second experimental set hardly induced liver damage, as confirmed by serum AST and ALT concentrations. Badger and colleagues reported that an HFD increased ALT levels, whereas an FED did not change the body weight and reduced ALT levels in rats (54). In humans, high-ALT levels were not associated with fat intake but rather were induced by an increase in sugar and carbohydrate intake. The patients who were enduring high-ALT levels also showed increased weight, body mass index, waist circumference, hip circumference, body fat density, and insulin resistance (55). Another research group showed that fat intake impaired the benefits of physical exercise in reducing lipid peroxidation, and it blocked the release of hepatic glutathione production for the muscles and the blood (5).

Our findings seem controversial in comparison with some previous reports on the protective effects of exercise on dietary fat-induced CVD (16–18). Instead of using moderate aerobic physical training like we successfully applied in both rat and mouse models against a life-threatening condition (24–26, 41), other authors used either high-intensity interval or resistance exercises (16–18). We should note that Boim and colleagues reported protective effects of exercise against hypertension and CVD risk when physically trained rats were treated with an aqueous extract of *Phalaris canariensis L* (56). Nevertheless, exercise could not reduce the cardiometabolic risk that was promoted by a high cholesterol diet in ApoE^−/−^ mice (28).

Hence, we suggest that increased intake of fat promotes metabolic and inflammatory changes that impair the protective effects of physical exercise against CVD development. This might illustrate that a healthy diet cannot be replaced solely by physical training to protect the cardiovascular system. Current findings warrant further investigation.

## Ethics Statement

All experiments followed the guidelines of the Committee on Care and Uses of Laboratory Animals of the National Research Council of the NIH (USA) and were approved by the Committee on Animal Research of the University of São Paulo (#163/2008 and #137/2010). Hence, male Wistar rats (*n* = 80; 60 days) were housed in plastic cages in a controlled room for light–dark cycles (12:12 h), temperature (24 ± 1°C), and humidity (60–70%). Rats had free access to water and standard chow and underwent adaptation to the new environment for 1 week before experiments started.

The ethical approved protocol #137/2010 was a multidisciplinary and multi-laboratory project coordinated by Prof. Dr. Sergio B. Garcia. Data from 6 out of 18 groups are reported herein. As requested by the Ethical Committee to not repeat animal experiments unnecessarily, some of our previous reports share few data from four groups: SED/STD [weight, cholesterol, TG, ALT, AST, visceral fat, hepatic MDA, and hepatic GSH; (32)]; SED/STD; STD/E20; and STD/E90 (cholesterol and TG) (24). These groups posed as control groups for carcinogen-exposed groups. Above all, current results are shown clearly, honestly, and without fabrication, falsification, or inappropriate data manipulation.

## Author Contributions

CF and SG designed the study; CF, KM, BG, JS, FF, AJ, JJ, DL, and FM performed experiments and analyses; CR, VK, and SG analyzed the data; CR, VK, SR, SU, and SG interpreted the data; CR, VK, SR, SU, and SG drafted the manuscript; CF, VK, KM, FF, AJ, JJ, BG, JS, DL, FM, SR, SU, and SG revised the manuscript critically for important intellectual content; CF, VK, KM, FF, AJ, JJ, BG, JS, DL, FM, SR, SU, and SG approved the final version of the manuscript submitted.

## Conflict of Interest Statement

The authors declare that the research was conducted in the absence of any commercial or financial relationships that could be construed as a potential conflict of interest.
