# Supplementary material for: High-Fat and Fat-Enriched Diets Impair the Benefits of Moderate Physical Training in the Aorta and the Heart in Rats
Source: Front Nutr. 2017 May 18;4:21. doi: 10.3389/fnut.2017.00021 (PMC5435813; doi:10.3389/fnut.2017.00021)
Supplement: Supplementary file 1 [file Table_1.DOCX]

**Table S1 | Design of the first statistical analysis for study I**

|  | **SED** | **E60** |
| --- | --- | --- |
| **STD** | **STD-SED *vs* STD-E60** | |
| **HFD** | **HFD-SED *vs* HFD-E60** | |

Standard diet (STD), high-fat diet (HFD), sedentary (SED), physical exercised for 60 min (E60).

**Table S2 | Design of the first statistical analysis for study II**

|  | **SED** | **E20** | **E90** |
| --- | --- | --- | --- |
| **STD** | **STD-SED *vs* STD-E20 *vs* STD-E90** | | |
| **HFD** | **HFD-SED *vs* HFD-E20 *vs* HFD-E90** | | |

Standard diet (STD), high-fat diet (HFD), sedentary (SED), physical exercised for 20 min (E20) and 90 min (E90).

**Table S3 | Design of the second statistical analysis for study I**

|  | **STD** | **HFD** |
| --- | --- | --- |
| **SED** | **STD-SED *vs* HFD-SED** | |
| **E60** | **STD-E60 *vs* HFD-E-60** | |

Standard diet (STD), high-fat diet (HFD), sedentary (SED), physical exercised for 60 min (E60).

**Table S4 | Design of the second statistical analysis for study II**

|  | **STD** | **HFD** |
| --- | --- | --- |
| **SED** | **STD-SED *vs* HFD-SED** | |
| **E20** | **STD-E20 *vs* HFD-E20** | |
| **E90** | **STD-E90 *vs* HFD-E90** | |

Standard diet (STD), high-fat diet (HFD), sedentary (SED), physical exercised for 20 min (E20) and 90 min (E90).
